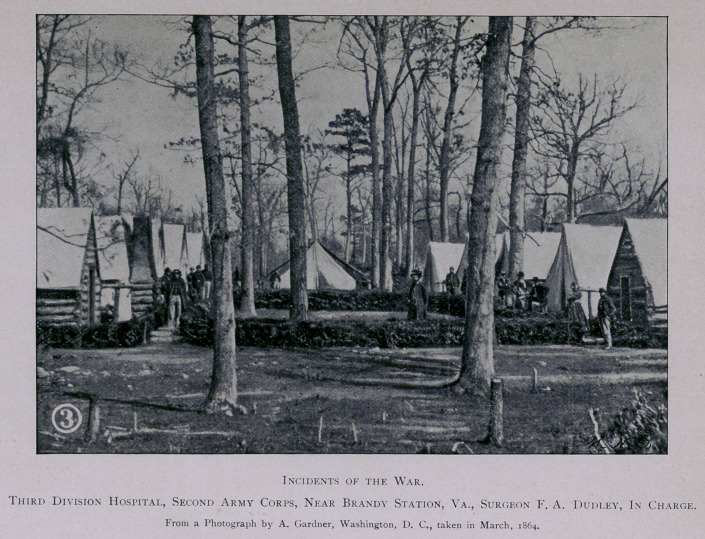# Reminiscences of Field-Hospital Service with the Army of the Potomac (Illustrated)1Concluded from the October number.

**Published:** 1889-11

**Authors:** William Warren Potter

**Affiliations:** Brevet Lieutenant-Colonel United States Volunteers; Surgeon in Charge First Division Field Hospital, Second Army Corps; Surgeon Fifty-seventh Regiment, New York Volunteers; Assistant Surgeon Forty-ninth Regiment New York Volunteers; Recorder Second Division Hospital in Sixth Army Corps, etc., etc.; 284 Franklin street


Buffalo Medical and Surgical Journal

Vol. XXIX. NOVEMBER, 1889. No. 4.

### Original Communications.

### REMINISCENCES OF FIELD-HOSPITAL SERVICE WITH THE ARMY OF THE POTOMAC.[1]

By WILLIAM WARREN POTTER,

Brevet Lieutenant-Colonel United States Volunteers; Surgeon in Charge First Division Field Hospital, Second Army Corps; Surgeon Fifty-seventh Regiment, New York Volunteers; Assistant Surgeon Forty-ninth Regiment New York Volunteers; Recorder Second Division Hospital in Sixth Army Corps, etc., etc.

This promotion afforded me an opportunity to pay a short visit home, my first absence from duty since entering the service. I was mustered in as surgeon at the War Department, in Washington, on the 22d of January, 1863, and reported to the regiment for duty on the 31st.

The 57th N. Y. Volunteers was then encamped above Falmouth, and was attached to the Third Brigade of the First Division of the Second Army Corps. This division was then commanded by Major-General W. S. Hancock, who subsequently became famous as commander of the Second Army Corps. I had known General Hancock when he was a brigade commander in General Smith's Division of the Sixth Corps, as well as the members of his personal staff, who were still with him, and this acquaintance served me to a good purpose in my new relations about to commence.

We remained in camp near Falmouth, the troops doing picket duty along the Rappahannock, until the Chancellorsville campaign opened April 27th.

On the morning of Tuesday, April 28th, we marched at sunrise, and on the 30th crossed the river at United States Ford, bivouacking near Chancellorsville late that night.

At the battle of Chancellorsville, May 1st to 4th, the hospital of the First Division, Second Corps, was located in the woods, three-fourths of a mile in the rear of the Chancellor House, near the road leading to United States Ford. Here it was impracticable to even

1. Concluded from the October number.

pitch the tents, for the position of the troops was so changeable, and the lines were so unstable that, besides the danger of the enemy's fire, there was the additional danger of possible capture; so the wounded were placed in rows upon blankets, the dry leaves gathered by the attendants serving in the place of straw. Colonel Nelson A. Miles, 61st N. Y. Volunteers (now Brevet Major-General U. S. A.), was brought into this hospital with a supposed mortal wound. He was placed upon the table for examination, and, while the surgeons were thus engaged, a shell burst near by, killing the ambulance sergeant who brought the gallant Colonel off the field, and who was sitting on his horse intently watching the surgeons, anxiously awaiting the result, that he might take back to the front accurate information concerning the condition of his beloved commander. The wound proved less serious than was at first supposed, though the symptoms of collapse were alarming; nevertheless, this distinguished officer was spared to render valuable service afterward, both with the Army of the Potomac, where he rose to the command of a division, and in fighting the Indians on the plains since the close of the civil war; his record as a soldier having passed into history, while he is yet in the full vigor of his usefulness. On Monday, May 4th, a train of ambulances was loaded with wounded, and sent across the Rappahannock at United States Ford, onwards to Potomac Creek Hospital. I was sent in charge of the train, and delivered the wounded at the hospital the same night, remaining there on duty for two weeks afterward. While en route we passed sufficiently near to witness Sedgwick's gallant fight at Banks's Ford,[1] the bursting of the shells above the tree-tops in the gray twilight, making a brilliant, though destructive, pyrotechnic display.

At Gettysburg the hospital of the First Division was literally shelled out of its first position. The site was chosen early in the day on the 2d of July, soon after the arrival of the corps on the field, after its night's march from Taneytown. In the afternoon, while there was yet quietude along the whole line, I rode over to General Meade's headquarters on the Taneytown road, and, after making a short call, passed on to Cemetery Hill, to take a survey of the field from that point. Sweeping my glass towards the left, I saw the Third Corps, under Sickles, advancing in magnificent line of battle towards the Emmetsburg Pike. The day had been cloudy, with a misty rain a portion of the time; but now the clouds were breaking away, and, as the sunlight glinted on the burnished muskets and bright colors of the advancing host, a most beautiful and entrancing picture was presented

1. The Sixth Corps here literally cut its way through the enemy, and crossed to the north bank in the night.—W. W. P.

to the view. Two general officers, Howard and Doubleday, were standing near by watching the scene intently, and when, presently, a white smoke was seen farther to the left, and the latter exclaimed, "There, General, go the enemy's batteries," I began to realize, indeed, that the battle had opened. Returning to my post, I called again at headquarters; but in a few minutes the shells began to fill the air with their shrieking and hissing music, the location being such as to receive all long-range and stray projectiles. The fire soon grew so hot that everybody took to horse—generals, staff officers, orderlies, and escort, all left the place, but in the most quiet manner—Meade for the front, Pleasanton to look after his cavalry, and other officers to their various posts of duty. Meanwhile, I discovered that the first position of our hospital had become untenable, by reason of being in range of the enemy's fire, and a new location covered in by a hill, near a stream of water, had been selected. Here we remained until the battle was over, performing operations and attending to the wounded night and day, until all were finally cared for and removed to more permanent hospitals. General S. K. Zook, of the First Division, received a mortal wound on the evening of July 2d, and we sent him to a house near by. He survived less than twenty-four hours, and it was my sad duty to minister to his sufferings during this period.

During the battle of Gettysburg, the hospitals of the army, excepting those of the Twelfth Corps, were without their usual camp equipage, and, as a consequence, everything had to be improvised as best it could. Houses, barns, straw-stacks, and all available localities were seized upon; while even woods were, in many instances, the only protection obtainable. It appears that General Meade had given strict orders that no wagons should go to the front, excepting the hospital and ammunition trains, but the Chief Quartermaster had somehow failed to include the hospital trains in the exception, hence the embarrassment. When this was finally discovered, it was too late to rectify the mistake, and so we were obliged to improvise, as I have stated. Thus it came about that during the greatest battle of the war—certainly a pivotal battle—the wounded were subjected to greater privations, in many respects, than when we were fighting on the soil, which, by common consent, was designated the enemy's country. But they made no complaint, and, as the weather was warm, the suffering by this deprivation of usual shelter was reduced to a minimum. The Twelfth Corps, which somehow succeeded in evading the order about the trains, brought its hospital wagons up, and was thus enabled to carry on its hospital work more systematically.

On the 8th of August, 1863, while the Second Corps was encamped near Morrisville, Va., guarding some of the fords of the Rappahannock, east of the Orange & Alexandria railroad, the following order was issued, assigning me to the charge of the First Division Hospital:

HEADQUARTERS SECOND ARMY CORPS,
August 8, 1863.

SPECIAL ORDERS,
No. 717.

Surgeon W. W. POTTER, 57th N. Y. Volunteers, is hereby detailed to the command of the hospital of the First Division, Second Corps, relieving Surgeon *George L. Potter*, who, on being thus relieved, will report to his regimental commander without delay.

By Order of BRIGADIER-GENERAL CALDWELL.

(Signed,) JOHN HANCOCK,
*Assistant Adjutant-General.*

I continued upon this duty until mustered out of service, and the remainder of this memoir will be devoted to an account of service in that capacity.

During the succeeding few months the army was engaged in a campaign of manœuvres, extending from Mitchell's Station back to Centerville, then out to the Rappahannock again; finally across the Rapidan to Mine Run, and thence back to Winter quarters, between those two rivers, with the headquarters of the army near Brandy Station. During this period, hospital work consisted chiefly in receiving and caring for the sick on the march, as we had comparatively few wounded to provide for, and we were practically an ambulance or flying hospital. In the retrograde movement to Centerville in October, however, the First Division, under General Caldwell, covered the rear the last day, October 14th, when there was some sharp work, culminating, just at nightfall, in the battle at Bristoe Station. During the day we were once or twice in precarious positions, our hospital train narrowly escaping capture at Auburn, in the early morning. I was obliged, also, on this occasion to provide for the cavalry wounded, besides my own, and, after the fight at Bristoe, all were taken to Centerville, where we arrived late at night. Being short of medical officers, I was compelled to make some urgent operations in the night, with only one surgical assistant; the hospital steward and nurses were, however, utilized to advantage, and all were cared for before morning. Next day all our wounded were sent to Fairfax Station for shipment to Washington, and we were again ready for the forward movement, which soon commenced.

All were cared for, did I say? No! Not all. One poor fellow, just returned from General Hospital, where he had been for months, was wounded that day by a shell, which shattered his right leg and left forearm at one fell swoop. He was placed in an ambulance and brought up to Centerville that night, but he was so low from shock that we dared not remove him therefrom, and so fed him with brandy and beef stock in the ambulance until morning, a nurse being specially detailed for that purpose. When daylight came he was still too feeble to go upon the operating table, and so was watched and fed until the order came to move in the afternoon of the 15th. Something now must be done, the order to move was imperative, and the wounded were all loaded into the ambulances, to go to Fairfax Station. Hastily summoning the Medical Director of the Corps, Dr. A. N. Dougherty, of Newark, N. J., now deceased, we determined, upon consultation, that the only proper way was to amputate. One ambulance was kept to receive this man, and the others were allowed to depart en train to the railroad station. A shower had now arisen, and all shelter had been struck and loaded in the wagons, so, while four men held a rubber blanket over us for protection from the rain, I made the double consecutive amputations of his right thigh and left arm, and placed him in the waiting ambulance with a special nurse and stimulants, to follow the remainder of the train to Fairfax. He recovered and wrote me afterward from General Hospital in Washington. His name is Frank Rose, private Co. D., 57th N. Y. Volunteers, and the case is recorded in the Medical and Surgical History of the War of the Rebellion,—the arm amputation in Part II., surgical volume, p. 711, and the thigh amputation in Part III., surgical volume, p. 253.

This incident is mentioned to show the exigencies of the service, and how even extreme surgical emergencies must be subordinated to the inexorable demands of military necessities; and, further, to show how, even under the most unpromising conditions and adverse circumstances, surgical work may turn to success,—how lives on the field were sometimes snatched from the very jaws of death.

At the Mine Run affair, in the last days of November, we only employed the ambulance hospitals in the Second Corps, as we had but few wounded, and they, for the most part, were only slight cases. The weather was bitter cold, and the only comfort to be derived from the movement was its brief duration. On our return to the north side of the Rapidan, every one felt that campaigning was over for the Winter, and we soon settled into the hum-drum ways of every day camp life. Orders were soon issued for the preparation of more permanent hospitals, and a site was selected for those of the Second Corps in a piece of woods situated about a mile from Brandy Station, on the road to Stevensburg. Trees were felled, ground cleared, and tents pitched for the three hospitals of the corps, which were arranged side by side in their numerical order, that of the First Division being on the right.

It so happened that within the lines of the First Division were two saw-mills, situated upon a stream that flowed along the camps, and which furnished the power to run them. They were immediately put in order by the Chief Quartermaster of the division, logs were cut and hauled by the soldiers, who enjoyed this diversion from ordinary military duties, and, by working the mills night and day, sufficient lumber was soon obtained to make the cantonments of the entire corps very comfortable. The hospitals received the first supply, next the enlisted men, and lastly, the officers; so that by the middle of January, 1864, the camps began to assume quite a home-like air.

The hospitals were laid out in streets, with a double row of tents on each side, facing inwards, and the quarters of the Surgeon-in-Charge located at the head of the street, facing south. A separate cot was provided for each patient in this wise: four crotched posts were driven into the ground, one at each of the four corners of the bed; a firm stick rested in the crotches across the head and foot, on which were placed small springy poles cut from straight saplings, extending lengthwise of the bed, and as close together as they could lie; a bed-sack filled with straw, a pillow, warm blankets, and clean white sheets, served to equip a very comfortable bed. The aisles, as well as the spaces between each cot, were floored; spacious fire-places were constructed in the rear end of each ward; and sidewalks built on both sides of the streets, and elsewhere about the camp, as convenience required. This may seem, as described, a crude and rough place for the care of the sick, to one not familiar with army life; but civilians, who visited these hospitals, were surprised and gratified to find them both cheerful and comfortable. It was, moreover, a matter of experience that recoveries were more prompt, not to say more certain, when the soldiers who were disabled by curable diseases, were treated in field hospitals, surrounded by comrades who had a personal interest in their welfare, and ministered unto by their own surgeons. The hygienic surroundings, too, were usually superior to those of large general hospitals, and, besides, the sick treated in tents have an incomparable advantage in being able to obtain plenty of fresh air without the dangers of a draught. [See illustrations.]

A special supply of fresh oysters, milk, and crackers, brought daily from Washington, together with other obtainable comforts and luxuries, contributed much to the welfare and contentment of the sick; while the presence of a bright, cheery, and faithful woman nurse,[1]

1. Miss Cornelia Hancock, who also rendered good service afterwards in the base hospitals at Fredericksburg and City Point. This deserving woman has rendered distinguished service on several occasions during dire disaster since the war—notably, at Charleston, S. C., after the earthquake, and more recently at Johnstown, Pa., after the flood.—W. W. P.

INCIDENTS OF THE WAR.

THIRD DIVISION HOSPITAL, SECOND ARMY CORPS, NEAR BRANDY STATION, VA., SURGEON F. A. DUDLEY, IN CHARGE.

From a Photograph by A. Gardner, Washington, D. C., taken in March, 1864.

who also presided over the special diet kitchen, aided not a little to make the service of the hospital more effectually successful. The wounded from Morton's Ford, February 6, 1864, instead of being sent to Washington after the first attention, were distributed to these three division hospitals, where all the operations were made, and where they were kept until recovery or other termination of the cases occurred.

Early in January, 1864, General Meade issued orders permitting officers who so desired, to invite their wives, mothers, or sisters, to visit the army for a limited period and something like 4,000 ladies availed themselves of this privilege, during the Winter and early Spring. A large music hall was built at General Caldwell's headquarters, (First Division, Second Corps,) which was in almost nightly use for concerts, hops, lectures, and other social gatherings. Grace Greenwood (Mrs. Lippincott) paid us a visit during the course of the season, and favored us with three or four of her characteristic "talks," which always bristled with wit, wisdom, and genuine loyalty. The frequent visits of many of these ladies to the hospitals, and their kind and cheery words to the sick, will long be remembered by both those who were the recipients and those who witnessed their beneficial effects, as a bright oasis in the desert-like expanse of war's dreadful arena.

In the latter days of April the unrecovered sick of the army were sent to Washington, surplus baggage and camp equipage sent to the rear, and everything put in readiness for an active campaign, which actually began on the 3d of May. The campaign equipment of the First Division Hospital consisted of twenty-two hospital tents, forty-three ambulances, fourteen army wagons to carry supplies, and five Autenreith medicine wagons. We had thirty-six hospital attendants under charge of a ward-master, a chief hospital steward, and a chief cook. Other hospital stewards, nurses, cooks, and attendants were supplied as occasion required.

The organization of the hospital staff, at the opening of the campaign, was as follows:

Surgeon W. W. POTTER, *57th N. Y. Volunteers*, In Charge.
Surgeon CHARLES S. HOYT, *39th N. Y. Volunteers*, Executive Officer.
Assistant Surgeon P. M. PLUNKETT, *2d Delaware Volunteers*, Recorder.
Lieutenant BURKHARDT, *66th N. Y. Volunteers*, Acting Assistant Commissary of Subsistence.

Dr. Plunkett was mustered out July 2, 1864, by reason of expiration of term of service, and his place was filled by the appointment of Assistant Surgeon J. C. Norris, 81st Pa. Volunteers. The division consisted of four brigades—one more than the usual number—and, consequently, our necessities were proportionately larger in the way of hospital equipment. Each brigade was allowed one medicine wagon of the Autenreith pattern, and I had one for my own operating uses, in which I also carried supplies to issue to the others in case of emergency, making five in all, as I have above stated.

During the Winter, Dr. Letterman had been, at his own request, relieved from duty as Medical Director of the Army of the Potomac, and Surgeon T. A. McParlin, U. S. A., appointed in his stead. Dr. McParlin proceeded to carry out the wise provisions of his predecessor's administration, and his orders at the opening of the campaign evinced a knowledge of the magnitude and responsibilities of his position, which gave him, at once, the confidence and support of the medical staff of the army, and which strengthened as the campaign progressed.

In the battle of the Wilderness, where we remained from the 4th to the 7th of May, we obtained native ice the first day, taken from an ice-house near the lines, which General Francis C. Barlow, then commanding the First Division, with his characteristic thoughtfulness for the welfare of his wounded, ordered seized and sent to the hospital. From this time until we reached the lines before Petersburg, we had liberal supplies of native ice wherever we established our hospital.

We moved towards Spottsylvania on the night of the 7th; were at Todd's Tavern on the 8th and 9th; at the River Po on the 10th; and at Spottsylvania from the 11th to the 19th. The hospital was located near Cossins's from the 11th to the 14th, where its capacity was taxed to the uttermost, more than 1,000 wounded having been received before noon of the 12th. General Barlow sent the Division band to the hospital on the 13th, to give a concert for the wounded, which cheered the men very much, as it was the first music we had been permitted to enjoy since crossing the Rapidan. The wounded of the Second Corps were sent to Fredericksburg on the 11th and 13th, numbering at both shipments 2,923, in 133 ambulances, and 258 army wagons. The First Division Hospital sent 450 on the 11th; and had still 950 for shipment on the 13th. We spent most of the night of the 13th in this work, and after exhausting all our transportation, both ambulances and army wagons, daylight found us with about 200 still on hand.[1]

1. This was, indeed, a most trying night, and the permanent staff could be seen, with lanterns in hand, superintending the loading of the wagons during all those weary hours, with the mud overfoot, and the rain still falling. I presume Dr. Hoyt, the then Executive Officer, who is now Secretary of the New York State Board of Charities, should his eye meet this, will remember the occasion vividly. General Francis A. Walker, Assistant Adjutant-General of the Second Corps, who was then an inmate of my own tent, as a guest for a week, by reason of an injury received on the morning of the assault at the salient, May 12th, will, I am sure, recall the scene.—W. W. P.

The movement of the corps to the left, during the night of the 13th, uncovered our position at Cossins's, and rendered a like movement of the hospital necessary; so we left the remaining wounded, supplied with the necessary medical officers, rations, and hospital supplies, to fall into the enemy's hands.[1] I left the place on the 14th, after completing all arrangements for their care, and soon after my departure the enemy's cavalry, under Rosser, came in, capturing all hospital attendants who wore no distinctive badge, and carrying off the greater part of the rations which had been left for the wounded. The Confederate wounded, who were left behind, were also removed to their own lines by the troopers. A force from the Second Corps was sent to drive away the marauders, but they were off before our troops arrived. Sadly enough, Surgeon Thomas Jones, 8th Pa. Reserves, left with the wounded of the Fifth Corps, similarly abandoned, was killed by one of our own soldiers, who, in the darkness, mistook him for a guerilla. On the 16th, just at evening, a train of ambulances, protected by Gibbon's Division, went to Cossins's and brought in the wounded from all the abandoned hospitals, together with the stores, tents, and attendants still remaining. After being fed and dressed, the wounded were sent to Fredericksburg.

At Cold Harbor, the Tyler House[2] on the left was first selected as our hospital site, but, as the military authorities deemed it unsafe, the tents were pitched in a field farther to the right, near army headquarters. Here we remained during the heaviest part of the battle, from the 2d to the 4th of June, where we cared for more than 1,000 wounded in our hospital. The dead bodies of three brigade commanders were brought to the First Division Hospital here, viz.: Colonel O. H. Morris, 66th N. Y.; Colonel H. B. McKeon, 81st Pa., and Colonel Peter A. Porter, 8th N. Y. H. Artillery, where they were embalmed and sent North. Colonel McKeon fell between the lines, and his body could not be recovered until after night-fall, when volunteers were called for, who brought it off.

On the 5th we moved the hospital to the Tyler House, where it was originally intended to establish it, the lines now having been sufficiently extended to protect that position. We were now about two miles from Gaines's mill, the scene of Porter's great battle of two years before, which was now within the enemy's line. Here the tents were pitched on a beautiful lawn facing an avenue of locusts of ancient lineage, leading from the road to the house, a distance of some twenty rods. The Recorder's office, hospital commissary, and

---

1. This was done under orders from army headquarters.—W. W. P.

2. The property of the late Dr. Tyler, a relative of ex-President Tyler. The owner died two years before, while the army under McClellan was occupying the Cold Harbor region.—W. W. P.

officers' mess were established in the house, which afforded conveniences for these important departments, so essential to the successful conduct of our hospital. A well-filled ice-house supplied us liberally with ice during the week we remained there, but it was completely emptied by the end of that time. Our mess was made up of the members of the permanent staff of the hospital, the five ambulance officers of the division, and the hospital commissary of subsistence; and during engagements I always invited the operating staff to join us, which they gladly did, as their own messes were temporarily disrupted. There was always a unity of feeling between the hospital staff and the ambulance officers in the First Division, and each department enjoyed the confidence and received the support of the other.

On Sunday, June 12th, preparations were made for the movement to the James river. The sick and wounded were sent to White House, the hospital packed up, and by night-fall we were on our way to participate in the plan recommended by McClellan in 1862, which the army was about to take up, namely, to attack Richmond and the rebel host from the South, via Petersburg. Fifteen ambulances and one medicine wagon accompanied each division; the remainder of the hospital train, consisting of ambulances, medicine wagons, and army wagons joining the supply trains and moving with them. The James river was crossed on a pontoon bridge, at Wilcox's landing, near Fort Powhatan.[1] The supply trains, having in some manner obtained the right of way over the bridge, delayed the crossing of the medical train from early morning until late in the afternoon of the 16th, so that it did not reach the front until between nine and ten o'clock that night. Meanwhile, a battle was in progress, the Second Corps having made an assault at six o'clock in the evening, and Medical Director Dougherty had selected sites for the hospitals; but nothing further could be done, excepting to prepare the ground and build arbors, until the wagons arrived. This was the first time during the campaign that the hospitals had not been fully prepared for the reception of wounded in advance of the necessity, and this was without fault of the medical department. Tents, however, were pitched, the wounded brought in, food prepared, and serious cases attended to; the hospital staff, ambulance corps, and attendants working hard all night with energy and alacrity for humanity's sake. By noon of the 17th, the First Division Hospital had received over 1,000 wounded, the third time this had happened in a single day since the opening of the campaign, the two other instances having been at Spottsylvania and Cold Harbor.

Mrs. General Barlow, who had been with us a few days at the

---

1. The river is nearly a mile in width at this point.—W. W. P.

Tyler House, visited us again at this point, and was kept busy in preparing milk-punch, which she administered to the wounded with her own hands. This philanthropic woman yielded up her life to her country's cause, breathing it out a month hence, from fatal disease contracted in her efforts to alleviate the suffering of wounded soldiers. The campaign thus far had been almost a perpetual battle from the Rapidan to Petersburg, taxing the energies and endurance of the medical staff and the ambulance service to an extreme degree; but they had not been found wanting, and, notwithstanding the unusual hardships of the campaign, had met every demand made upon them in a spirit of cheerful obedience to duty, and with a promptitude amidst many difficulties that were, oftentimes, well-nigh overwhelming. I would not wish to make, at this time, invidious distinctions where all worked so well and faithfully, but a few men, besides the hospital staff proper, were a tower of strength to me during this trying campaign. The never-tiring and always amiable Medical Director of the corps, Dr. A. N. Dougherty, was ever ready with advice and timely aid; General Francis C. Barlow, a martinet in discipline and a brave and capable field commander, always manifested the kindliest interest in the hospital, granting every request consistent with military exigency to render it efficient; Dr. D. H. Houston, Surgeon-in-Chief; Dr. J. W. Wishart, Chief Operator, and Dr. A. Vander Veer, Surgeon of the 66th N. Y. Volunteers, were among my confidential advisers and able coadjutors in the work. These, and others whom I should be glad to mention by name, did space permit, contributed in an inestimable degree to whatever of success my administration of the First Division Hospital may have attained.

The operations before Petersburg soon began to partake of the nature of a siege, and the hospitals, likewise, began to assume a more permanent mien. At the Burchard House, on the Norfolk & Petersburg stage road, where the First Division Hospital located itself sometime in July, ovens were built, cots erected, and many of the comforts of Winter quarters provided. Purchases of fresh fruits, vegetables, eggs, milk, etc., were made from the hospital fund; ice was obtained from City Point, and many other luxuries were likewise added to the ordinary army supplies that served to improve the efficiency of our work. A portion of the family, with a few servants, still occupied the house and premises, but Mr. Burchard himself, who was the father-in-law of the Confederate General Dearing, was absent in Petersburg, whither he went with Mrs. Dearing just before our forces appeared. The establishment of our lines on the 16th of June cut off his return, and he was thus compelled to remain away until Petersburg fell, in April, 1865.

Several expeditionary movements were made by the Second Army Corps, while the Army of the Potomac was besieging Petersburg. One of the first of these was to Deep Bottom, on the James river, which began on the evening of July 26th, and which had for its objective the diversion of a sufficient force of the enemy away from his works to enable the Ninth Corps to make an assault, with increased chances of success, after the explosion of its famous mine, that was now about completed. Another object in the movement was to attack Richmond from the north side of the James river, in case it should be found feasible to do so. The first division took fifteen ambulances on this expedition, and sufficient material to conduct a flying hospital. We crossed the James river before dawn, on the 27th, and captured a battery of four twenty-pounder Parott guns at sunrise. We remained in observation until the dark of the 29th, when our wounded were shipped by steamer, and the corps returned to the Petersburg lines in time to witness the explosion of the mine on the morning of the 30th, though, happily, none of our troops were engaged in the assault.

We reëstablished our hospital at the Burchard house, where we remained until the 12th of August, when we once more moved to Deep Bottom. The hospital train crossed the river on the same bridges as before, and established itself on the north bank. Here we remained from the 14th to the 20th of August, during which time there was much spirited fighting, and we had many wounded to care for, among whom was Captain James C. Bronson, 57th New York Volunteers. Captain Bronson was doing staff duty with the Third Brigade, when he received a wound of the right forearm that rendered amputation necessary. I cared for him in my own quarters from the 14th to the 20th, when we were ordered to ship our wounded by steamers, and again prepare to retrace our steps to our old position. The morning of August 21st found us once more at the Burchard house, but we moved the same day to the left, to assist Warren's Fifth Corps in securing the Weldon railroad, and finally brought up, on the 25th, at Reams' Station. The 26th found us still again at the Burchard house; and this time we remained comparatively quiet for some time. The movement to Hatcher's Run, in October, temporarily disrupted the hospital, but it was again established at the Burchard house, and substantially occupied that site during the late Fall and Winter, even until the final campaign, in the early Spring of 1865.

Colonel James A. Beaver,[1] 148th Pa., who lost a leg at Reams' Station, August 25th, was quartered in this house until his recovery. After the amputation, which was made in the temporary hospital on

1. Afterward Brevet Brigadier-General U. S. Volunteers, and now Governor of Pennsylvania.—W. W. P.

the field, he was brought hither on a stretcher, a distance of over eight miles, by a detail of sixteen men.[1] Here he was faithfully nursed by Miss Gilson, niece of the Hon. Mr. Fay, of Chelsea, Mass., a prominent member of the United States Sanitary Commission. Mr. Fay, himself, was also a frequent visitor to the hospital, contributing time and means to the alleviation of the suffering sick and wounded, here and elsewhere throughout the army.

Both the Sanitary and Christian Commissions rendered efficient service to the hospital inmates during the overland campaign, as well as at other times, furnishing supplies of lemons, oranges, shirts, drawers, and other useful articles, besides rendering personal attention to individual cases, writing and mailing letters, and doing a variety of other work which cannot be specified, but which economized the time of the already overworked medical staff and attendants, on many occasions.

The large depot hospitals established at Fredericksburg, Port Royal, White House, and City Point, under the charge of Surgeon E. B. Dalton, U. S. Volunteers, were an important part of the hospital system of the Army of the Potomac, and are worthy of more than a passing reference; so, too, of the railway and steamboat transportation of the wounded; but this article has already reached its limits, and the distinctive features of field-hospital service which was its purpose to present, have already been set forth.

From the 3d of May to the 19th of September, 1864, the First Division Hospital register contained the names of between 7,000 and 8,000 sick and wounded, who had been cared for during that period. Many of these were slight cases that recovered in a few days, more or less, and were returned to duty. If they had been at once sent beyond the Field Hospital to the General Hospitals, in the North, they would not have returned for several months, if at all. In this way, alone, the division system of hospital organization exhibited an economic value of no mean proportions.

The post of the surgeon in action has erroneously been supposed by many to be free from danger. The records of the Surgeon General's office, at Washington, show that during the late war thirty-two medical officers were killed in battle, or by guerrillas, or partisans; nine killed by accident; and eighty-three wounded in action, of whom ten died. This is believed to be a casualty list proportionately larger than that of any other staff corps. Three medical officers were killed at the battle of Antietam, one of whom, Surgeon W. J. H. White,

1. A similar service was rendered to General Sickles, at Gettysburg, the same night of the amputation, July 2d. A detail of forty soldiers carried him to Westminister, a distance of twenty-five miles. I have no doubt both these lives were saved by this expedient.—W. W. P.

U. S. A. Medical Director of the Sixth Corps, was the first officer killed in Franklin's command in that battle. On the 10th of May, 1864, at the engagement at the River Po, Surgeon A. N. Dougherty, U. S. Volunteers, Medical Director of the Second Corps, than whom there was no braver or more efficient officer in the medical staff of the army, was wounded by a shell while superintending the affairs of his department. These instances are cited to show that even medical officers of the higher grades were often exposed to the greatest dangers.

As an interesting final fact, showing the importance of the care of the sick and wounded of an army from another standpoint, it is ascertained from carefully compiled financial tables, also derived from the Surgeon General's office, that the total money cost of the maintenance of the Medical Department of the army from 1861 to 1865, exclusive of the salaries of officers, was something over forty-seven and one-third millions of dollars. Surely a republic that dealt so generously with its soldiers who suffered from the casualties of war in its defense, cannot justly be charged with neglectfulness, indifference, or ingratitude.

284 FRANKLIN STREET.

## Figures and Tables

**Figure f1:**
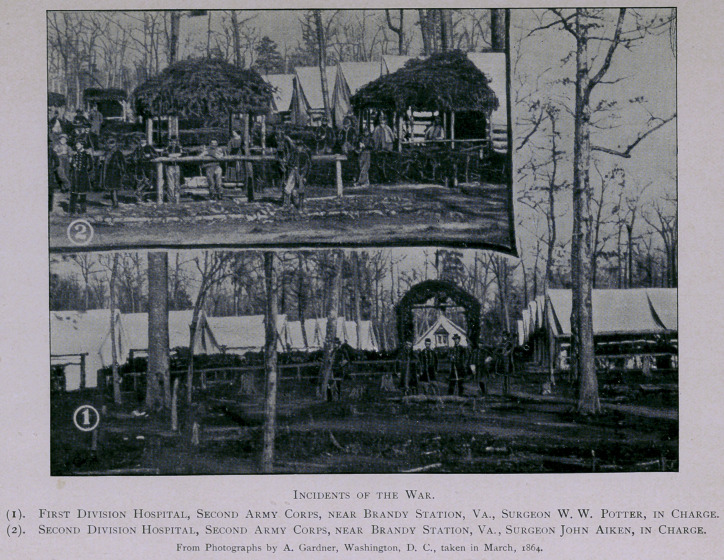


**Figure f2:**